# The Association between Self-Reported Difficulties in Emotion Regulation and Heart Rate Variability: The Salient Role of Not Accepting Negative Emotions

**DOI:** 10.3389/fpsyg.2017.00328

**Published:** 2017-03-09

**Authors:** Endre Visted, Lin Sørensen, Berge Osnes, Julie L. Svendsen, Per-Einar Binder, Elisabeth Schanche

**Affiliations:** ^1^Department of Clinical Psychology, University of BergenBergen, Norway; ^2^Division of Psychiatry, Haukeland University HospitalBergen, Norway; ^3^Department of Biological and Medical Psychology, University of BergenBergen, Norway; ^4^Bjørgvin District Psychiatric Centre, Haukeland University HospitalBergen, Norway; ^5^Bjørgvin District Psychiatric Centre, Knarvik, Haukeland University HospitalIsdalstø, Norway

**Keywords:** emotion regulation, Heart Rate Variability (HRV), emotion regulation strategies, acceptance, Difficulties in Emotion Regulation Scale (DERS), self-report scales

## Abstract

Difficulties in emotion regulation are associated with development and maintenance of psychopathology. Typically, features of emotion regulation are assessed with self-report questionnaires. Heart rate variability (HRV) is an objective measure proposed as an index of emotional regulation capacity. A limited number of studies have shown that self-reported difficulties in emotion regulation are associated with HRV. However, results from prior studies are inconclusive, and an ecological validation of the association has not yet been tested. Therefore, further exploration of the relation between self-report questionnaires and psychophysiological measures of emotional regulation is needed. The present study investigated the contribution of self-reported emotion regulation difficulties on HRV in a student sample. We expected higher scores on emotion regulation difficulties to be associated with lower vagus-mediated HRV (vmHRV). Sixty-three participants filled out the Difficulties in Emotion Regulation Scale and their resting HRV was assessed. In addition, a subsample of participants provided ambulatory 24-h HRV data, in order to ecologically validate the resting data. Correlation analyses indicated that self-reported difficulties in emotion regulation was negatively associated with vmHRV in both resting HRV and 24-h HRV. Specifically, when exploring the contribution of the different facets of emotion dysregulation, the inability to accept negative emotions showed the strongest association with HRV. The results are discussed and need for future research is described.

## Introduction

Emotions are multifaceted phenomena involving both experiential, behavioral and somatic domains (Gross, 2015a). Thus, a person that is experiencing an emotion like anger or sadness, will experience rapid changes in how he or she feels and behaves, in addition to bodily reactions. There has been increasing attention on how individuals orchestrate emotional reactions in order to adapt to an ever changing environment. Emotion regulation is now considered to play a central role in psychological health and well-being, and has been proposed as a transdiagnostic factor in mental health (Gross and Muñoz, 1995; Fernandez et al., 2016). Although the concept of emotion regulation have gained increased popularity, controversy and debate regarding the operationalization of the concept is still ongoing, as is evident by the many definitions of the concept (Bloch et al., 2010). In clinical research, the definition of emotion regulation by Gratz and Roemer (2004) is frequently used. These authors propose that emotion regulation involves multiple processes. First, emotion regulation consists of the ability to be aware of emotions as they arise, and to experience them clearly. Emotional awareness and clarity thus enable us to monitor and evaluate emotional experience, and to modify the experience when needed. It has been suggested by several authors that deficiencies in emotional awareness and clarity may lead to emotion regulation failure (Gross, 2015b). Second, emotion regulation involves the ability to accept emotions. The inability to accept emotional reactions, by suppressing or avoiding them, is associated with increased emotional valence, cognitive load and physiological arousal (Hayes et al., 2006). Further, acceptance represent a psychological flexibility which is increasingly being incorporated into modern psychotherapy (Dryden and Still, 2006). Third, emotion regulation is also associated with the ability to tolerate emotions to meet situational demands and personal goals without being overwhelmed by them. For example, when experiencing negative emotions, behaving in accordance with ones goals, selecting appropriate emotion regulation strategies, and modulating emotional responses to meet goals and situational demands is of high importance to adapt to the environment.

To measure emotion regulation and the above mentioned facets, Gratz and Roemer (2004) constructed the Difficulties in Emotion Regulation Scale (DERS). Converging evidence show that self-reported difficulties with emotion regulation as measured by the DERS are associated with psychopathology including borderline personality disorder (Rajappa et al., 2011), post-traumatic stress disorder (Tull et al., 2007) as well as mood and anxiety disorders (Mennin et al., 2009; Fowler et al., 2014; Menezes and Bizarro, 2015).

When assessing complex, multifaceted processes like emotion regulation, several methods of assessment is of interest to gain more insight into different aspects of emotion regulation, such as psychophysiological assessments (Seeley et al., 2015). As self-report questionnaires may measure the subjective experience of emotion regulation, psychophysiological assessment may be valid assessment of the somatic processes underlying emotional processing. Furthermore, psychophysiological assessments can provide data that are not prone to biases that underlies the use of self-report measures, including social desirability, misunderstanding of items and deficient self-knowledge or psychological mindedness. In addition, some populations may not be able to provide self-reports, making it important to assess validity of other modes of assessments. For example, previous research have utilized the measurement of cortisol levels in toddlers to assess stress (Drugli et al., 2017), skin conductance in postoperative patients to assess pain perception (Storm, 2008) and autonomic flexibility in patients with alcohol related brain damage to assess quality of life (Steinmetz et al., 2016). Further, given the usage of the DERS in clinical populations, it is also of importance to validate this self-report measure to other assessments that propose to measure similar or related concepts.

Heart rate variability (HRV) is a proposed psychophysiological marker of emotion regulation capacity (Appelhans and Luecken, 2006; Thayer et al., 2012). The heart is innervated by the central autonomic network (Benarroch, 1993), through both postganglion sympathetic fibers and vagally mediated parasympathetic nervous system (PNS). This network allows the individual to adapt in a flexibly manner to a continuously changing environmental demands (Kemp and Quintana, 2013). The vagal innervation slows down the heart rate, whereas the sympathetic nervous system (SNS) increases the heart rate – often in situations with perceived threats or danger. According to the Theory of Neurovisceral Integration the variability between heart beats is affected by several brain areas such as the prefrontal cortex, a brain area also involved in regulating emotions (Thayer and Lane, 2009; Thayer et al., 2012). With emotional arousal, e.g., emotional stress responses, the SNS produces higher heart rates when there are difficulties in emotion regulation, the PNS may not slow down this heart rate and the physiological stress responses. The dominance of the SNS on heart rate will therefore lead to a lower HRV (Appelhans and Luecken, 2006). High HRV in a resting state indicates a flexible PNS, and conversely, low HRV indicates sympathetic and more inflexible influence of the heart rate. The ability to adjust the physiological systems to meet environmental demands thereby reflects a general ability to adapt to situational contingencies. There is emerging evidence that lower HRV is associated with psychological disorders, including anxiety (Chalmers et al., 2014), depression (Kemp et al., 2010; Kemp et al., 2012), schizophrenia (Montaquila et al., 2015), and borderline personality disorder (Koenig et al., 2016). Vagus mediated HRV (vmHRV) is thus used as a peripheral measure of prefrontal inhibition of the amygdala (Thayer et al., 2012). As the prefrontal cortex is crucial in the inhibition of the amygdala and is considered a neural basis of emotion regulation, vmHRV can be defined as a peripheral measure of emotion regulation (Johnstone and Walter, 2014). Evidence also indicates that general emotion regulation capacity is associated with vmHRV, including acceptance of emotions (O’Connor et al., 2002; Cristea et al., 2014), and accessibility of emotion regulation strategies (Aldao and Mennin, 2012; Gillie et al., 2015). In addition, we have previously shown that higher self-reported usage of the emotion regulation strategy self-compassion is associated with higher vmHRV (Svendsen et al., 2016). In sum, available research points to vmHRV being a marker of emotion regulation capacity (Appelhans and Luecken, 2006; Beauchaine and Thayer, 2015). However, the relation between vmHRV and self-report measures of emotion regulation difficulties is inconclusive, and few studies have yet explored this association.

To our knowledge, only two studies have investigated the association between self-reported difficulties in emotion regulation and HRV. One of these studies showed that a subsample of participants reporting higher emotion regulation difficulties had lower HRV and recovered slower in their heart rates after a negative emotion elicitation using film clips, compared to participants reporting lower emotion regulation difficulties (Berna et al., 2014). Thus, participants reporting high emotion regulation difficulties also had lower HRV, and needed more time to recover after an emotional provocation. However, the study did not find a significant correlation between self-reported emotion regulation and HRV when using the whole sample. The study used a shorter, non-validated version of the DERS, which may explain the insignificant finding. In contrast, Williams et al. (2015) used the full and validated version of the DERS and found an association between HRV and several facets of emotion regulation difficulties as measured by DERS. More specifically, they found that lower resting HRV was negatively correlated with all but one facet of emotion regulation difficulties (emotional awareness). Multiple hierarchial regression analyses showed that only impulse control difficulties and emotional clarity were associated with resting HRV. The latter analysis controlled for the effect of emotional stress such as trait anxiety and rumination. Emotional dysregulation is related to higher stress reactions and as such, controlling for trait anxiety and rumination may remove variance that is due to difficulties in emotion regulation. As a recent studies have shown, worry is highly associated with self-reported difficulties in emotion regulation (Allan et al., 2015), as well as lower HRV and lower usage of the adaptive emotion regulation strategy reappraisal (Knepp et al., 2015). Further, higher usage of the maladaptive emotion regulation strategy rumination may reflect limited access to emotion regulation strategies. In healthy adults, it can therefore be argued that it is important to measure self-reported emotion dysregulation with the DERS without adjusting for overlapping emotional traits. Further, both of the aforementioned studies exclusively relied on laboratory derived data, raising questions about the ecological validity of the results.

The main aim of the present study was to further investigate the association between self-reported difficulties in emotion regulation and vmHRV. We also wished to investigate the validity of the findings using 24-h ambulatory HRV data. Assessing vmHRV throughout 24-h using ambulatory equipment in naturalistic settings may validate data retrieved from shorter laboratory settings (Ewing et al., 1984). These long-term assessments may also shed light on the importance of diurnal fluctuations in vagal activity (i.e., Brosschot et al., 2007). Our first hypothesis was that higher self-reported emotion regulation difficulties would be associated with HRV. Given that there are few studies that have explored this association, our study was exploratory without specific hypotheses on which facets of emotion regulation that would be associated with HRV. Our second hypothesis was that similar results will be found using 24-h ambulatory HRV data.

## Materials and Methods

### Participants

Participants were recruited from the student population of the University of Bergen, Norway through internal announcements to the student population by email and information about the study on a university web-page directed at students. The current study was a pilot study conducted to investigate aspects of mindfulness training and its association with psychophysiological measures considered to be included in a larger planned randomized controlled study. One study containing data from a subsample of this study was published elsewhere (Svendsen et al., 2016). Exclusion criteria were history of severe psychiatric illness, heart condition, use of sedative or psychoactive medication, and previous experience with mindfulness- or acceptance based interventions or retreats. In total, 62 participants were enrolled. Two participants were removed from the analyses due to poor quality on ECG-recordings. In sum, data from 60 participants were analyzed (70% female, age range 19 – 31, mean age 23.5, *SD* = 2.6). A random subsample of 34 participants wore Actiheart for 24-h inter-beat-interval (IBI) data. Data from six participants were excluded from further analysis due to poor data quality, resulting in a subsample of 28 participants (60.7% female, age range 19–31, mean age 23.7, *SD* = 2.7). No outliers were found after visual inspection of scatter plots in DERS and HRV data. The protocol was approved by the local ethics committee, and all participants gave written consent in accordance with the Helsinki declaration.

### Procedure and Measurement

Prior to assessment, participants were asked not to exercise or consume nicotine or caffeine-preparations 3 h before the collection of data. Moreover, participants were required to refrain from use of alcohol or psychoactive drugs the day prior to assessment. Participants were informed about the measurements that followed, but no information was given regarding the hypotheses of the study. Participants were seated in a room where they filled out self-report questionnaires, including questionnaires not reported in the current paper. For collection of HRV data, all participants were seated during the electrocardiogram (ECG) recording. Cardiac activity data was recorded with a lead-II ECG at a 1000 Hz sampling rate using a Biopac 4.0 BS (Biopac Systems, Inc., Santa Barbara, CA, USA). Resting HRV was assessed within a 15-min period. ECG data was recorded approximately at the same time in the afternoon, to avoid potentially confounding circadian effects (Bonnemeier et al., 2003). To control for the potential confounding variable of Body Mass Index (BMI), self-reported height and weight data was collected to calculate BMI.

After self-report and ECG-data were obtained, a 24-h ambulatory acquisition device was placed on a random subsample of the participants, and this device was collected at the same time the following day. The participants in the sub-sample were asked to refrain from consuming alcohol or other psychoactive drugs while HRV data were acquired. Twenty-four hour HRV was acquired with Actiheart monitors (Cambridge Neurotechnology, Cambridge, UK), a device that have shown to give a reliable account of NNI data (Brage et al., 2005). The Actiheart recorder was placed at two adhesive Ag/AgCl ECG electrodes (T815 Dia. 55) below the apex of sternum, midway below the V1 and V2 positions, and horizontally toward as laterally as possible.

#### Heart Rate Variability Analysis

In the resting HRV data, R-spikes in the ECG signal were identified through the algorithm in Kubios version 2.0 software (Tarvainen et al., 2014), and later subjected to visual inspection. In the few cases where the R-tacks were misplaced by the automated QRS detection, this was corrected manually. Trend components were removed with the smoothness priors detrending method (λ = 500). Then, variability between successive R-spikes (Normal to normal intervals (NNIs)) was obtained from ECK recordings to calculate HRV. The root mean square of successive differences (RMSSD), measured in milliseconds, and High Frequency Power HRV in absolute values of power (ms^2^; HF HRV), defined as 0.14–0.4 Hz in the frequency band were calculated using the Kubios software. We chose to use the RMSSD component in our main analyses due to replication purposes (Williams et al., 2015) but most importantly because it is considered to be mediated by the vagus nerve, and therefore indicating PNS activity (Appelhans and Luecken, 2006; Shaffer et al., 2014). However, the HF HRV was used to investigate whether this component yielded the same results as the RMSSD (Task Force of the European Society of Cardiology the North American Society of Pacing Electrophysiology, 1996). The resting HF HRV correlated highly with RMSSD (*r* = 0.935, *p* < 0.000; full correlation matrix including resting HF HRV is included in Supplementary Table 1). Finally, high frequency peak values (HF Peak) were obtained as a measure of respiration frequency, in order to control for possible bias by respiration, as was done in a similar analysis (Williams et al., 2015). *Post hoc* analyses showed that controlling for respiration did not affect the main results (results not shown here).

The 24-h NNI data was imported into Actiheart commercial software (Actiheart software 2.132^1^) and the manufacturer’s algorithm applied to clean and interpolate noisy and missing heart rate data (Cambridge Neurotechnology Ltd.; Brage et al., 2005). In line with current recommendations, any R-R interval lesser than 400 ms and larger than 2000 ms was excluded from the HRV analyses (Task Force of the European Society of Cardiology the North American Society of Pacing Electrophysiology, 1996). The 24-h RMSSD of the HRV data were calculated with the software Kubios version 2.0 (Tarvainen et al., 2014). In addition to obtaining the total 24-h HRV, the data was segmented into daytime and nighttime HRV. The segmentation was based on chronological time, as nighttime was defined as the time from 24.00 to 08.00, and daytime was defined as the time from 08.00 to 16.00. The Actiheart monitors provided a simplified actigraph, and all analyzed samples showed low activity during the defined nighttime.

#### Self-report Questionnaire

Difficulties in emotion regulation were assessed using the DERS (Gratz and Roemer, 2004). DERS is a 36 item questionnaire designed for measuring six facets of difficulties in emotion regulation. The items are rated on a five-point scale ranging from 1 [almost never (0–10%) to 5 (almost always (91–100%)]. The facets include (with Chronbach’s alpha from the present study presented within parentheses): (1) Lack of acceptance of emotions (DERS NON-ACCEPT, e.g., “When I’m upset, I feel ashamed with myself for feeling that way.”, Chronbach’s α = 0.91); (2) Difficulties engaging in goal-directed behavior (DERS GOALS, e.g., “When I’m upset, I have difficulty focusing on other things.”, Chronbach’s α = 0.94); (3) Impulse control difficulties (DERS IMPULSE, e.g., “When I’m upset, I feel out of control.”, Chronbach’s α = 0.91); (4) Lack of emotional awareness (DERS AWARENESS, e.g., “I pay attention to how I feel.”, Chronbach’s α = 0.76); (5) Limited access to emotion regulation strategies (DERS STRATEGIES, e.g., “When I’m upset, I believe that I will remain that way for a long time.”, Chronbach’s α = 0.92); (6) Lack of emotional clarity (DERS CLARITY, e.g., “I am confused about how I feel.”, Chronbach’s α = 0.88). Higher score on DERS yield more difficulties in emotion regulation. DERS showed excellent internal consistency in the current investigation (Chronbach’s α = 0.95). DERS was translated into Norwegian and validated in an Norwegian sample by Dundas et al. (2013). Recent studies have confirmed DERS’ acceptable internal consistency, construct validity and factor structure (Fowler et al., 2014; Ritschel et al., 2015).

Body Mass Index (BMI) was calculated using self-reported height and weight data.

### Statistical Analysis

All statistical tests were conducted using SPSS version 23. All resting RMSSD and HF HRV in addition to the 24-h RMSSD, daytime RMSSD and nighttime RMSSD scores were log transformed to approximate normal distribution and used in consequent analyses and tests (logRMSSD, logHF, 24-h logRMSSD, daytime logRMSSD, nighttime logRMSSD. First, bivariate correlations were computed to investigate the relationship between DERS, resting logRMSSD, and 24-h logRMSSD, daytime logRMSSD and nighttime logRMSSD. Bivariate correlations between all variables and logHF can be found in the Supplementary Table 2. Second, multiple hierarchical regression analyses were conducted to investigate the prediction of DERS on resting logRMSSD. Step one included potential covariates known to affect HRV, including age (Reardon and Malik, 1996), gender (Koenig and Thayer, 2016), BMI (Koenig et al., 2013) and respiration index (HF Peak; e.g., Beda et al., 2007); step two included DERS total and subscales, respectively. Due to expected multicollinearity, we ran independent regression analyses for each DERS scale. Finally, in order to ecologically validate our resting HRV data, we conducted bivariate correlations between the DERS subscales and 24-h HRV data, controlling for age, BMI and gender. To control for these covariates, standardized residual scores of 24-h logMRSSD, daytime logRMSSD and nighttime logRMSSD were calculated in a linear regression analysis, so that the variance explained by age, BMI and gender were extracted. All analyses were two-tailed and were analyzed using a set level of significance of *p* < 0.05. In addition, we adjusted for multiple analyses by using Bonferroni correction of alpha level in the hierarchical regression analyses (p.05/6), which gives an alpha-corrected *p* level of 0.008. We report on both levels of significance, due to the explorative nature of the study thus avoiding making Type II error (Perneger, 1998).

## Results

No difference between males and females were found in respect to all HRV variables. A paired *t*-test showed that nighttime HRV was significant higher than daytime HRV (*t*_27_ = -4.01, *p* < 0.000). Descriptive data from all participants (*n* = 60) and subsample (*n* = 28) including means (*M*), standard deviations (*SD*) can be found in **Table 1**. Bivariate correlations showed that only the DERS Total (*r* = -0.273, *p* = 0.035) and the DERS Non-accept (*r* = -0.345, *p* = 0.007) correlated significantly with resting logRMSSD of the DERS scores. Thus, participants that had lower resting vmHRV reported higher difficulties in emotion regulation and higher difficulties in accepting negative emotions. In addition, resting logRMSSD correlated with 24-h logRMSSD (*r* = 0.491, *p* = 0.008), daytime logRMSSD (*r* = 0.374, *p* = 0.05) and marginally significantly with nighttime logRMSSD (*r* = 0.372, *p* = 0.051). None of the DERS scales correlated significantly with the long-term logRMSSD data when not controlling for covariates of sex, gender and BMI (see **Table 2**).

**Table 1:** Means (M) and standard deviations (SD) and differences between females and males on all variables.

	All participants (*n* = 60)	Female (*n* = 42)	Male (*n* = 18)	*p*
Age	23.53 (2.62)	23.55 (2.60)	23.50 (2.75)	0.95
BMI	21.71 (2.28)	21.69 (2.38)	21.78 (2.11)	0.88
resting logRMSSD	1.72 (0.22)	1.74 (0.20)	1.67 (0.27)	0.37
HF peak	0.21 (0.05)	0.21 (0.05)	0.22 (0.06)	0.66
DERS Total	90.77 (25.70)	94.00 (25.01)	83.22 (26.40)	0.15
DERS Non-accept	14.98 (5.72)	15.45 (5.33)	13.89 (6.57)	0.38
DERS Goals	16.40 (5.50)	17.29 (5.29)	14.33 (5.55)	0.07
DERS Impulse	14.08 (6.27)	15.19 (6.49)	11.50 (5.00)	0.02
DERS Aware	15.28 (4.57)	15.41 (4.29)	15.00 (5.29)	0.78
DERS Strategies	18.25 (7.45)	18.86 (7.13)	16.83 (8.19)	0.37
DERS Clarity	11.25 (3.97)	11.24 (3.48)	11.28 (5.04)	0.98

	**Subsample (*n* = 28)**	**Female (*n* = 17)**	**Male (*n* = 11)**	***p***

24-h logRMSSD	1.76 (0.13)	1.78 (0.15)	1.73 (0.11)	0.25
Daytime logRMSSD	1.74 (0.16)	1.74 (0.40)	1.72 (0.32)	0.67
Nighttime logRMSSD	1.87 (0.17)	1.88 (0.43)	1.85 (0.30)	0.72


*BMI, Body Mass Index; logRMSSD, log-transformed Root Mean Square of Successive Differences; DERS, Difficulties in Emotion Regulation Scale.*

**Table 2:** Correlation matrix of all variables.

Variable	1	2	3	4	5	6	7	8
(1) Resting logRMSSD	-							
(2) DERS Total	-0.27*	-						
(3) DERS Non-accept	-0.35**	0.86***	-					
(4) DERS Goals	-0.23	0.77***	0.55***	-				
(5) DERS Impulse	-0.15	0.86***	0.67***	0.68***	-			
(6) DERS Awareness	-0.01	0.33*	0.20	-0.01	0.12	-		
(7) DERS Strategies	-0.22	0.88***	0.77***	0.67***	0.78***	0.02	-	
(8) DERS Clarity	-0.24	0.73***	0.61***	0.44***	0.50***	0.46**	0.50***	-
**Subsample (*n* = 28)**								
24-h logRMSSD	0.49**	-0.28	-0.23	-0.15	-0.16	-0.24	-0.21	-0.17
Nighttime logRMSSD	0.37	-0.21	-0.10	-0.10	-0.10	-0.23	-0.19	-0.15
Daytime logRMSSD	0.37*	-0.35	-0.36	-0.21	-0.23	-0.20	-0.23	-0.29


*DERS, Difficulties in Emotion Regulation Scale; logRMSSD, log-transformed Root Mean Square of Successive Differences. ^∗^*p* < 0.05; ^∗∗^*p* < 0.01; ^∗∗∗^*p* < 0.001.*

Similar results were evident using the HF HRV component (see Supplemental Table 1).

In order to assess the relative contributions of self-reported emotion regulation difficulties on resting logRMSSD, we conducted independent multiple hierarchial regression analyses using DERS Total in addition to the six subscales as dependent variables and resting logRMSSD as independent variable. The analyses shared a first step, including age, gender, BMI and respiration index values (HF Peak) as covariates. The second step included DERS Total and the subscales as predictor variables respectively. Multiple hierarchial regression analyses showed that variance in resting logRMSSD was explained by 9% from the DERS Total score (β = -0.31, *t* = -2.48, *p* = 0.016). Thus, there is a significant association between lower vmHRV and higher self-reported emotion regulation difficulties when covariates are controlled for.

Multiple hierchial regression analyses using DERS Non-accept as a predictor variable in the second step showed that DERS Non-accept explained 12.6% of the variance (β = -0.37, *t* = -3.02, *p* = 0.004). Thus, having difficulties with accepting negative emotional reactions is associated with lower vmHRV, even when controlled for covariates, and alpha corrigated for number of analyses. DERS Goals explained 9.1% of the variance in resting logRMSSD (β = -0.31, *t* = -2.5, *p* = 0.015). The remaining subscales showed no significant models using multiple hierarchial regression analyses. In all analyses, no significant effect of the covariates gender, BMI and age on the resting logRMSSD were found. When applying the HF HRV into the model as dependent variable, similar results emerged on DERS Total (β = -0.25, *t* = -1.96, *p* = 0.055), DERS Non-accept (β = -0.34, *t* = -2.7, *p* = 0.008) and DERS Goals (β = -0.23, *t* = -1.8, *p* = 0.081). Thus, when utilizing HF HRV into the model, only DERS Non-accept remains significant. Model summaries of the multiple hierarchial regression analysis, including model change statistics are shown in **Table 3**. The complete regression matrix is enclosed in Supplementary Table 2.

**Table 3:** Hierarchial regression analysis examining self-reported difficulties in emotion regulation as a predictor of vmHRV (*N* = 60).

Step and predictor variable	*B*	SE B	β	*R*^2^	Δ*R*^2^
Step 1, all models:				0.127	0.127
Age	-0.001	0.011	-0.008		
Gender	-0.054	0.061	-0.113		
BMI	-0.001	0.013	-0.015		
PeakHF	-1.462	0.573	-0.334		
Step 2, model 1:				0.216	0.09^∗^
DERS Total	-0.003	0.001	-0.309^∗^		
Step 2, model 2:				0.253	0.126^∗∗†^
DERS Non-accept	-0.014	0.005	-0.369^∗∗†^		
Step 2, model 3:				0.217	0.091^∗^
DERS Goals	-0.013	0.005	-0.312^∗^		
Step 2, model 4:				0.163	0.037
DERS Impulse	-0.007	0.005	-0.204		
Step 2, model 5:				0.127	0.001
DERS Awareness	-0.001	0.006	-0.025		
Step 2, model 6:				0.182	0.055
DERS Strategies	-0.007	0.004	-0.24		
Step 2, model 7:				0.172	0.046
DERS Clarity	-0.012	0.007	-0.219		


*DERS, Difficulties in Emotion Regulation Scale. ^∗^*p* < 0.05. ^∗∗^*p* < 0.01. ^†^*p* < 0.008 (Significant after Bonferroni correction). Complete regression matrix of the covariates Age, Gender, BMI and PeakHF can be found in the Supplementary Table 2.*

Finally, to examine the ecological validity of our findings, bivariate correlation analyses using long-term logRMSSD controlling for age, gender and BMI, showed that the DERS total score correlated significantly with 24-h logRMSSD (*r* = -0.38, *p* = 0.049) and daytime logRMSSD (*r* = -0.40, *p* = 0.33), but not nighttime logRMSSD (*r* = -0.24, *p* = 0.218). In addition DERS Non-accept correlated with daytime logRMSSD (*r* = -0.45, *p* = 0.017). The 24-h, daytime and nighttime logRMSSD variables did not correlate significantly with the remaining DERS subscales.

## Discussion

The purpose of the present study was to explore the associations between a well-established measurement of emotion regulation (DERS) and HRV, a proposed psychophysiological marker of emotion regulation capacity. Results showed that higher self-reported difficulties in emotion regulation were negatively associated with resting vmHRV in our bivariate correlation and multiple hierarchical regression analyses. When exploring the individual subscales of DERS, difficulties with accepting negative emotions, in addition to the inability to act in accordance with personal goals when experiencing negative emotions were associated with resting vmHRV. Not accepting negative emotions had the strongest association with resting vmHRV, and this association remained significant after Bonferroni adjusting the alpha level for number of analysis. Higher self-reported difficulties in emotion regulation were also negatively associated with vmHRV measured in a 24-h ambulatory setting. More specifically, we found that daytime HRV were negatively associated with both the total score of the DERS, as well as the non-acceptance subscale when controlling for the covariates of gender, sex and BMI. In contrast, no associations were found between nighttime vmHRV and the DERS in the same analyses. In sum, our hypotheses of vmHRV to reflect self-reported emotion regulation capacity was supported both in resting laboratory derived data and in the ambulatory data collected through 24-h.

The results indicate that self-reported difficulties with emotion regulation, and especially not accepting emotions, can be detected by measuring the variability between heart beats. The results give support to the model of Neurovisceral Integration (Thayer and Lane, 2009), that higher self-reported difficulties in emotion regulation and acceptance of negative emotions are associated lower variability between consequent heart beats. Thus, the findings indicate that emotion regulation capacity is related to the ability to flexibly modulate physiological processes in response to changing contexts.

Our findings confirms the hypothesis that self-reported emotion regulation difficulties are associated with autonomic flexibility as measured by HRV, and thus partially replicate earlier results (Williams et al., 2015). In addition, our findings from the 24-h measurement of HRV indicate that the results obtained from the short term HRV are ecologically valid. However, our findings were different in terms of associations between facets of emotion regulation difficulties and vmHRV. Whereas Williams et al. (2015) found that particularly lack of emotional clarity and impulse control were associated with resting vmHRV, we found non-acceptance of negative emotional reactions to be the strongest predictor of vmHRV. From a Neurovisceral Integration theoretical perspective (Thayer and Lane, 2000), the difficulty of accepting negative emotional reactions is associated with limited cortical inhibition of the amygdala. The ability to attain an attitude of acceptance and willingness to experience negative emotions is considered preventive in regards to development of psychopathology. Aldao et al. (2010) showed in their meta-analysis that acceptance of emotions is negatively associated with symptoms of anxiety and depression. In contrast, avoiding negative feelings and pushing them away is associated with psychopathology (Hayes et al., 2006) and decreased positive emotional experience and increased amygdalae and sympathetic activation (Gross, 2015a). Interestingly, acceptance as an emotion regulation strategy can be considered as an antecedent emotion regulation strategy that can be applied before an emotional reaction (i.e., accepting fear-related aspects of a situation) (Werner and Gross, 2010), in addition to a response-focused emotion regulation strategy that can be applied after an emotional reaction has occurred (i.e., accepting feeling afraid). In contrast, suppression as an emotion regulation strategy is considered as purely response focused emotion regulation strategy (Gross and John, 2003). The literature suggest that acceptance requires less cognitive resources to regulate emotions compared to suppression (Chambers et al., 2009; Lutz et al., 2014). Thus, by being both an antecedent and a response-focused emotion regulation strategy, acceptance may enable the person be more flexible in meeting potential emotionally charged situations. It is interesting that this type of psychological flexibility is associated with flexibility of the autonomic nervous system, here represented by HRV. Thus, acceptance of emotional responses may be an important focus for psychological treatment for psychopathology. Interestingly, the ability to accept negative emotions is addressed through third-wave cognitive therapies like Mindfulness-Based Cognitive Therapy for depression (Segal et al., 2013), and Compassion-Focused Therapy (Gilbert, 2009).

Although the findings of the present study support the hypothesis that self-reported difficulties in emotion regulation is associated with vmHRV, several limitations should be mentioned. First, our sample was consisting of relatively young healthy students with a majority of females, which may limit the ecological validity of the results. Although the present study adds support to the notion that increased emotion dysregulation is reflected in parasympathetic flexibility, the underlying facets of emotion dysregulation may differ across age. It could be hypothesized that hard working students that want to be included in a study offering a mindfulness-based intervention are less accepting of their emotional reactions. Also considering the sample, we did not assess the participants’ mental health, i.e., conducting semi structured interviews to confirm that the sample was a non-clinical sample. Another limitation is the use of one single questionnaire to assess emotion regulation. Although the DERS is a common inventory in assessing emotion regulation it may not capture the whole complexity of the concept. Finally, we did not perform follow-up studies to assess the reliability of our findings in this sample.

Consequently, future research should use other samples across age and clinical diagnoses to further expand our understanding of self-reported emotion dysregulation and vmHRV. Furthermore, longitudinal experimental studies should assess self-reported difficulties in emotion regulation and vmHRV to investigate temporal changes in these variables as result of psychological interventions. Also, studies should continue to use 24-h recordings of vmHRV on larger samples to obtain statistical significant results. Finally, the association between acceptance and HRV should further be investigated, for example in experimental settings, where participants are instructed to accept or suppress emotions.

Despite the limitations, the present study is a valuable contribution to the literature on emotion regulation difficulties, as it indicates that self-report measurement of these difficulties have reverberations into our bodily reactions, including central and peripheral somatic markers.

## Author Contributions

The following authors have contributed to the enclosed manuscript: EV. Project planning, collection of data, analyses of data, main author of manuscript: written the abstract, introduction, methods and materials and discussion. LS: Project planning, collection of data, analyses of data, supervision of statistical analyses, co-author and supervisor in writing process. BO: Project planning, collection of data, analyses of data (HRV), supervision of statistical analyses, supervision in writing process. JS: Project planning, collection of data. P-EB: Project planning, collection of data. ES: Project planning, collection of data, supervision in writing process.

## Conflict of Interest Statement

The authors declare that the research was conducted in the absence of any commercial or financial relationships that could be construed as a potential conflict of interest.

## Footnotes

1 http://www.camntech.com
