# Supplementary material for: The Association between Self-Reported Difficulties in Emotion Regulation and Heart Rate Variability: The Salient Role of Not Accepting Negative Emotions
Source: Front Psychol. 2017 Mar 9;8:328. doi: 10.3389/fpsyg.2017.00328 (PMC5343522; doi:10.3389/fpsyg.2017.00328)
Supplement: Supplementary file 1 [file Table_1.docx]

| Supplemental table 1 |  |  |  |  |  |  |  |  |  |
| --- | --- | --- | --- | --- | --- | --- | --- | --- | --- |
| Correlation matrix of all variables. | |  |  |  |  |  |  |  |  |
| Variable | 1. | 2. | 3. | 4. | 5. | 6. | 7. | 8. | 9. |
| 1. Resting vmHRV RMSSD | - |  |  |  |  |  |  |  |  |
| 2. Resting vmHRV HF | .94*** | - |  |  |  |  |  |  |  |
| 3. DERS Total | -.27* | -.21 | - |  |  |  |  |  |  |
| 4. DERS Nonaccept | -.35** | -.31* | .86*** | - |  |  |  |  |  |
| 5. DERS Goals | -.23 | -.15 | .77*** | .55*** | - |  |  |  |  |
| 6. DERS Impulse | -.15 | -.11 | .86*** | .67*** | .68*** | - |  |  |  |
| 7. DERS Awareness | -.01 | -.01 | .33* | .20 | -.01 | .12 | - |  |  |
| 8. DERS Strategies | -.22 | -.16 | .88*** | .77*** | .67*** | .78*** | .02 | - |  |
| 9. DERS Clarity | -.24 | -.21 | .73*** | .61*** | .44*** | .50*** | .46** | .50*** | - |
|  |  |  |  |  |  |  |  |  |  |
| Subsample (n=28) |  |  |  |  |  |  |  |  |  |
| 24-h vmHRV RMSSD | .49** | .48** | -.28 | -.23 | -.15 | -.16 | -.24 | -.21 | -.17 |
| Daytime logRMSSD | .37* | .33 | -.21 | -.10 | -.10 | -.10 | -.23 | -.19 | -.15 |
| Nighttime logRMSSD | .37 | .32 | -.35 | -.36 | -.21 | -.23 | -.20 | -.23 | -.29 |
| Note. DERS: Difficulties in Emotion Regulation Scale; vmHRV: vagal mediated Heart Rate Variability.  *p < 0.05; **p < 0.01; ***p < 0.001. | | | | | | | | |  |

| Supplemental table 2 | | |  |  |  |  |  | |  |
| --- | --- | --- | --- | --- | --- | --- | --- | --- | --- |
| Hierarchial regression analysis examining self-reported difficulties in emotion regulation as | | | | | | | | | |
| a predictor of vmHRV (N = 60). Complete regression matrix of the covariates Age, Gender, | | | | | | | | | |
| BMI and PeakHF. | |  |  |  |  |  | |  |  |
|  |  |  |  |  |  |  | |  |  |
| Step and predictor variable | | |  | B | SE B | β | | R^2^ | ΔR^2^ |
| Step 1, all models: | |  |  |  |  |  | | 0.127 | 0.127 |
| Age |  |  |  | -0.001 | 0.011 | -0.008 | |  |  |
| Gender |  |  |  | -0.054 | 0.061 | -0.113 | |  |  |
| BMI |  |  |  | -0.001 | 0.013 | -0.015 | |  |  |
| PeakHF |  |  |  | -1.462 | 0.573 | -0.334* | |  |  |
| Step 2, model 1: | |  |  |  |  |  | | 0.216 | .09* |
| Age |  |  |  | -0.003 | 0.010 | -0.038 | |  |  |
| Gender |  |  |  | -0.083 | 0.059 | -0.173 | |  |  |
| BMI |  |  |  | -0.005 | 0.012 | -0.054 | |  |  |
| PeakHF |  |  |  | -1.467 | 0.548 | -0.336** | |  |  |
| DERS Total | |  |  | -0.003 | 0.001 | -0.309* | |  |  |
| Step 2, model 2: | |  |  |  |  |  | | 0.253 | .126**† |
| Age |  |  |  | -0.001 | 0.010 | -0.013 | |  |  |
| Gender |  |  |  | -0.076 | 0.057 | -0.159 | |  |  |
| BMI |  |  |  | -0.010 | 0.012 | -0.103 | |  |  |
| PeakHF |  |  |  | -1.440 | 0.535 | -0.329** | |  |  |
| DERS Nonaccept | |  |  | -0.014 | 0.005 | -0.369**† | |  |  |
| Step 2, model 3: | |  |  |  |  |  | | 0.217 | .091* |
| Age |  |  |  | -0.001 | 0.010 | -0.006 | |  |  |
| Gender |  |  |  | -0.091 | 0.060 | -0.190 | |  |  |
| BMI |  |  |  | 0.000 | 0.012 | 0.000 | |  |  |
| PeakHF |  |  |  | -1.545 | 0.548 | -0.353** | |  |  |
| DERS Goals | |  |  | -0.013 | 0.005 | -0.312* | |  |  |
| Step 2, model 4: | |  |  |  |  |  | | 0.163 | .037 |
| Age |  |  |  | -0.003 | 0.011 | .0,037 | |  |  |
| Gender |  |  |  | -0.081 | 0.062 | -0.168 | |  |  |
| BMI |  |  |  | -0.005 | 0.013 | -0.049 | |  |  |
| PeakHF |  |  |  | -1.474 | 0.566 | -0.337* | |  |  |
| DERS Impulse | |  |  | -0.007 | 0.005 | -0.204 | |  |  |
| Step 2, model 5: | |  |  |  |  |  | | 0.127 | 0.001 |
| Age |  |  |  | -0.001 | 0.011 | -0.009 | |  |  |
| Gender |  |  |  | -0.055 | .0.61 | -0.114 | |  |  |
| BMI |  |  |  | -0.001 | 0.013 | -0.014 | |  |  |
| PeakHF |  |  |  | -1.463 | 0.578 | -0.335* | |  |  |
| DERS Awareness | |  |  | -0.001 | 0.006 | -0.025 | |  |  |
| Step 2, model 6: | |  |  |  |  |  | | 0.182 | 0.055 |
| Age |  |  |  | -0.003 | 0.011 | -0.030 | |  |  |
| Gender |  |  |  | -0.068 | 0.060 | -0.143 | |  |  |
| BMI |  |  |  | -0.005 | 0.012 | -0.053 | |  |  |
| PeakHF |  |  |  | -1.490 | 0.560 | -0.341* | |  |  |
| DERS Strategies | |  |  | -0.007 | 0.004 | -0.24 | |  |  |
| Step 2, model 7: | |  |  |  |  |  | | 0.172 | 0.046 |
| Age |  |  |  | -0.004 | 0.011 | -0.46 | |  |  |
| Gender |  |  |  | -0.054 | 0.059 | -0.113 | |  |  |
| BMI |  |  |  | -0.002 | 0.012 | -0.025 | |  |  |
| PeakHF |  |  |  | -1.390 | 0.564 | -0.318* | |  |  |
| DERS Clarity | |  |  | -0.012 | 0.007 | -0.219 | |  |  |
| Note. DERS: Difficulties in Emotion Regulation Scale. *p < .05. **p < .01 | | | | | | | | |  |
| † p < .008 (Significant after Bonferroni correction) | | | | |  |  |  | |  |
